# Anchor Detection Strategy in Moderated Non-Linear Factor Analysis for Differential Item Functioning (DIF)

**DOI:** 10.1177/01466216251401206

**Published:** 2025-11-24

**Authors:** Sooyong Lee, Suyoung Kim, Seung W. Choi

**Affiliations:** 1 Wisconsin Center for Educational Research, The University of Wisconsin-Madison, Madison, WI, United States; 2 Crown Family School of Social Work, Policy, and Practice, 278762 University of Chicago, Chicago, IL, US; 3 Educational Psychology, 12330 The University of Texas at Austin, Austin, TX, US

**Keywords:** MNLFA, differential item functioning, anchor detection, constrained-baseline, information criteria

## Abstract

Ensuring measurement invariance is crucial for fair psychological and educational assessments, particularly in detecting Differential Item Functioning (DIF). Moderated Non-linear Factor Analysis (MNLFA) provides a flexible framework for detecting DIF by modeling item parameters as functions of observed covariates. However, a significant challenge in MNLFA-based DIF detection is anchor item selection, as improperly chosen anchors can bias results. This study proposes a refined constrained-baseline anchor detection approach utilizing information criteria (IC) for model selection. The proposed three-step procedure sequentially identifies potential DIF items through the Bayesian Information Criterion (BIC) and Weighted Information Criterion (WIC), followed by DIF-free anchor items using the Akaike Information Criterion (AIC). The method’s effectiveness in controlling Type I error rates while maintaining statistical power is evaluated through simulation studies and empirical data analysis. Comparisons with regularization approaches demonstrate the proposed method’s accuracy and computational efficiency.

To ensure comparability across observations, measurements must yield precise results that reflect the intended construct (e.g., language proficiency; Camilli & Shepard, 1994). In other words, scaled scores must remain uninfluenced by extraneous factors unrelated to the underlying trait being measured, such as group membership. With this condition met, equivalently scaled scores can be meaningfully compared across individuals and groups, a principle rooted in measurement invariance (Millsap & Everson, 1993). Differential Item Functioning (DIF) is a key method to evaluate this, determining whether scores reflect the intended construct with unintended biases (Lord, 1980, p. 220; Millsap, 2011). DIF occurs when individuals with equal ability but differing characteristics (e.g., gender and race) respond differently to items, potentially distorting conclusions about ability (Martiniello, 2009). Identifying DIF is thus vital for preserving validity and fairness in assessment (Kim & Yoon, 2011).

Moderated Non-linear Factor Analysis (MNLFA) offers a flexible framework for assessing measurement invariance (Bauer & Hussong, 2009). By allowing model parameters to vary with covariates, MNLFA enables detection of both uniform and non-uniform DIF across multiple DIF sources (e.g., Coxe & Sibley, 2023). Conceptually, it combines the strengths of the Multiple Indicators Multiple Causes (MIMIC) model and Multiple Group (MG) analysis. MIMIC models are particularly advantageous for accommodating various types of DIF sources simultaneously, while MG models offer the flexibility to capture heterogeneous factor variances across groups (Bauer, 2017). MNLFA, in essence, integrates these capabilities into a unified framework, making it well-suited for complex data structures.

A central challenge in DIF analysis is selecting anchor items—those that function equivalently across groups and serve as a baseline for scaling (Kopf et al., 2015). Existing methods include iterative procedures (Choi et al., 2011; Chun et al., 2016; Hidalgo-Montesinos & Lopez-Pina, 2002), regularization (Belzak & Bauer, 2020; Magis et al., 2015), or graphical tests (Yuan et al., 2021). However, research on how to effectively incorporate anchor selection within the MNLFA framework is limited.

While some efforts have explored regularization-based anchor selection under the MNLFA framework (Bauer et al., 2020), these mainly focus on dichotomous data. The extension to polytomous data, commonly used in psychological testing, has yet to be fully realized. Because MNLFA is a relatively recent methodology compared to more established approaches such as MG analysis or MIMIC models, less attention has been devoted to methodological issues surrounding anchor detection. Given its rising use in applied research (Behrens et al., 2025; Stevens et al., 2022), a tailored anchor strategy for polytomous data is urgently needed to fully leverage MNLFA’s potential in DIF detection.

This study proposes an anchor detection strategy for MNLFA applied to polytomous data, especially in the context where anchor items are not predefined. The method iteratively identifies DIF-free items and integrates this step into MNLFA estimation. An exploratory simulation study evaluated the method under conditions that mirror practical applications. Two research aims guided this work: (1) test the proposed anchor selection procedure within MNLFA and (2) assess its performance against existing methods (e.g., regularization) in terms of anchor recovery (hit rates), Type II error, Type I error, power, and precision of DIF effect. The method was also applied to empirical data to demonstrate its practical utility.

## Moderated Non-linear Factor Analysis With Graded Response Model

In recent years, Moderated Non-linear Factor Analysis (MNLFA) has gained increasing attention in psychometrics as a powerful approach for assessing measurement invariance across both categorical and continuous covariates. By integrating the strengths of the MIMIC model and the MG model, MNLFA overcomes their respective limitations in detecting DIF (Bauer et al., 2020).

The MIMIC model supports simultaneous testing of DIF from binary or continuous DIF sources (e.g., gender or age), with factor mean differences (impact) considered. However, it assumes homogeneous factor variance across DIF sources, potentially biasing estimates of non-uniform DIF (Lee et al., 2024). In contrast, the MG model accommodates group-specific factor means and variances, enhancing its ability to detect DIF across discrete groups (Thissen et al., 1993), but is limited when DIF is influenced by continuous covariates.

MNLFA integrates MG flexibility into the MIMIC framework (Bauer, 2017; Curran et al., 2014), enabling item intercepts, loadings, means, and variances to be moderated by DIF sources, whether categorical or continuous. This enables comprehensive DIF detection while accounting for impact effects on both factor means and variances.

### Measurement Model for Ordinal Responses

For ordinal response data, the Graded Response Model (GRM; Samejima, 1969), defines cumulative probability functions, $P_{c}^{+}\left(\eta \right)$ which specify the probability of responding in category c or higher as:

(1) $$P_{ijc}^{+}\left(\eta \right)=\log\left(\frac{P\left(y_{ij}\geq c|\eta_{i}\right)}{P\left(y_{ij}<c|\eta_{i}\right)}\right)=\lambda_{j}\eta_{i}-\tau_{jc},$$

(2) $$\eta_{i}=\mu +\varepsilon_{i},\varepsilon_{i}\;∼\;N\left(0,\sigma^{2}\right),$$

where $P_{ijc}^{+}\left(\eta \right)$ is a cumulative probability that examinee i ’s response to item j falls in category c or above, given latent trait $\eta_{i}$ ; $\tau_{jc}$ is the threshold parameter^1^ (i.e., item intercept) for category *c* of item *j*, and $\lambda_{j}$ is the factor loading parameter (i.e., discrimination or item slope) for item *j*. The latent trait $\eta_{i}$ is assumed to follow a normal distribution with a mean of μ and residual variance $\sigma^{2}$ ; typically, μ=0 and $\sigma^{2}=1$ , for identification.

The probability of responding in category c of item j is then defined by the difference between the cumulative probability of a response to that category or higher and the cumulative probability of a response in category ( c+1 ) or higher (Samejima, 1969):

(3) $$P_{ijc}\left(\eta \right)=P_{ijc}^{+}\left(\eta \right)-P_{ij\left(c+1\right)}^{+}\left(\eta \right)$$

which can be written as:

(4) $$P_{ijc}\left(\eta \right)=\left\{ \begin{array}{ll} 1-P_{ijc}^{+}\left(\eta \right), & if{\;c}_{i}=0\\ P_{ijc}^{+}\left(\eta \right)-P_{ijc+1}^{+}\left(\eta \right), & if\;0<c_{i}<m-1\\ P_{ijc+1}^{+}\left(\eta \right), & if{\;c}_{i}=m-1 \end{array}\right.$$

### MNLFA Model Specification

Under the MNLFA framework, the key parameters of GRM—thresholds ( τ **)**, factor loadings ( λ **)**,factor mean ( E(η) or μ ) and factor variance (V(η) or $\sigma^{2}$ )—are modeled as functions of exogenous covariates (Bauer & Hussong, 2009). Given *p* covariates, **X**, these parameters are specified as a linear function and a log-linear function, respectively, as follows:^2^

(5) $$\tau_{c}=\tau_{c0}+ΒX$$

(6) $$\lambda =\lambda_{0}+\Gamma X$$

(7) $$E\left(\eta \right)=\omega_{0}+\Omega X$$

(8) $$V\left(\eta \right)=\exp\left(\kappa_{0}+ΚX\right)$$

where $\tau_{c0}$ and $\lambda_{0}$ are *j ×* 1 vectors representing the baseline thresholds and item slopes, respectively, when all X=0 . Matrices B and Γ (of size *j × p*) contain elements $\beta_{\text{jp}}$ and $\gamma_{\text{jp}}$ , which capture uniform and non-uniform DIF effects associated with a set of *p* DIF sources. Ω reflects the mean differences in η across levels of **X** (impact). Similarly, ${Κ}_{j}$ captures variance differences in η across **X**. Baseline parameters $\omega_{0}$ and $\kappa_{0}$ are fixed at zero for model identification when all item intercepts and slopes are freely estimated.

### DIF in MNLFA

With this specification, DIF can be tested across *p* continuous covariates or categorical grouping variables. Uniform DIF for item *j* is assessed by the significance of $\beta_{jp}$ in Β , after accounting for group-level differences in latent means via Ω . Non-uniform DIF is indicated by $\gamma_{jp}$ , allowing item slopes to vary across covariates, while controlling for latent variance differences via Κ . Items not regressed on covariates are treated as DIF-free anchors.

## Anchor Detection Approach

MNLFA is increasingly used for evaluating measurement invariance across categorical and continuous covariates (Behrens et al., 2025; Stevens et al., 2022). Its flexibility makes it well-suited for psychological and educational assessments. However, anchor item detection, especially with polytomous items, remains underexplored, despite being critical for accurate DIF detection (Finch, 2005; Wang et al., 2012). Improper anchors can bias item parameters and distort group comparisons.

Earlier methods ranged from likelihood ratio tests to iterative DIF procedures (Candell & Drasgow, 1988; Hidalgo-Montesinos & Lopez-Pina, 2002; Kopf et al., 2015). Recent advances, like Lasso regularization, offer promise for joint DIF and anchor detection (Magis et al., 2015; Wang et al., 2023), though most studies focus on dichotomous items (Belzak & Bauer, 2020), leaving polytomous cases less studied.

MIMIC-based anchor methods offer a theoretical basis for adaptation in MNLFA (e.g., Lopez-Rivas et al., 2009; Wang, 2004). Chun et al. (2016) compared constrained-baseline, free-baseline, and sequential-free-baseline methods. The constrained-baseline approach begins by designating all items as anchors. This method is straightforward, yet it has significant limitations, particularly inflated Type I error rates, rendering it unreliable for anchor detection (Stark et al., 2006). The free-baseline approach starts with a single anchor item and iteratively adds items. The sequential-free-baseline approach is a combination of the two aforementioned approaches, which initially applies constrained methods for anchor detection before switching to free-baseline testing for the remaining items. The free-baseline approach and the sequential free-baseline approach outperformed the constrained-baseline approach, which exhibited significantly inflated Type I errors (Chun et al., 2016).

Although the free- and sequential free-baseline approaches may be promising for MNLFA, our preliminary analyses suggest that they are computationally prohibitive within this framework. The constrained-baseline approach remains more computationally feasible for MNLFA, but its known issue of inflated Type I errors necessitates methodological improvement. This limitation led us to develop a refined approach that retains computational efficiency while addressing the issue of Type I error inflation.

## Refined Constrained-Baseline Approach for MNLFA

We propose a refined constrained-baseline approach using information criteria (ICs) rather than likelihood ratios.

### Information Criteria for Model Selection

Information criteria, such as Akaike Information Criterion (AIC), Bayesian Information Criterion (BIC), and Weighted Information Criterion (WIC), are frequently used in model selection in item response theory (IRT) and structural equation modeling (SEM) (e.g., Lin et al., 2017; Robitzsch, 2022). These criteria balance model fit and complexity to guard against overfitting or underfitting, making them particularly suitable for anchor detection.

AIC is defined as AIC = −2LL+2k , where LL is the log-likelihood and k is the number of estimated parameters, minimizing information loss and prioritizing predictive accuracy. In large samples, the −2LL dominates, making the penalty term ( 2k ) relatively negligible. This leads AIC to prefer more complex, less parsimonious models, potentially flagging DIF effects even when they are not truly present (Magis et al., 2015; Vrieze, 2012).

BIC, defined as = −2LL+k×log⁡(N) , adheres to Bayesian principles and consistently identifies the true model as sample size grows (Preacher & Yaremych, 2023). Its stronger penalty ( k×log⁡(N)) for complexity helps avoid overfitting and favors simpler models. This property makes BIC particularly useful for identifying the true model when it exists within the candidate set (Bollen et al., 2014). However, its conservativeness can increase Type II errors in small samples (Dziak et al., 2020; Vrieze, 2012).

Given the trade-offs between AIC and BIC, researchers have investigated the WIC as a middle-ground solution. WIC aims to balance the trade-off between AIC’s efficiency and BIC’s consistency by facilitating data-driven weighting of the two criteria (Magis et al., 2015). It is especially useful in DIF detection, where neither AIC nor BIC alone may provide the optimal balance between sensitivity and specificity in identifying DIF items.

A key limitation of IC is their lack of established thresholds for meaningful differences. Unlike traditional hypothesis testing, IC comparisons don’t provide significance levels, meaning even small IC gaps can influence model selection. This implies that even a marginally smaller IC value could favor a more complex model (with fewer degrees of freedom or modeled DIF effects) over a more constrained model (with greater degrees of freedom or no DIF effects).

This study proposes a structured approach to systematically assess IC differences. The fundamental premise of our approach is that models with true DIF effects will exhibit larger IC differences compared to the baseline model than models without DIF effects. These differences can be evaluated using outlier detection methods from regression analysis, while employing robust standard errors to account for non-normality in the IC differences.

### Refined Constrained-baseline Approach

**Figure 1.**
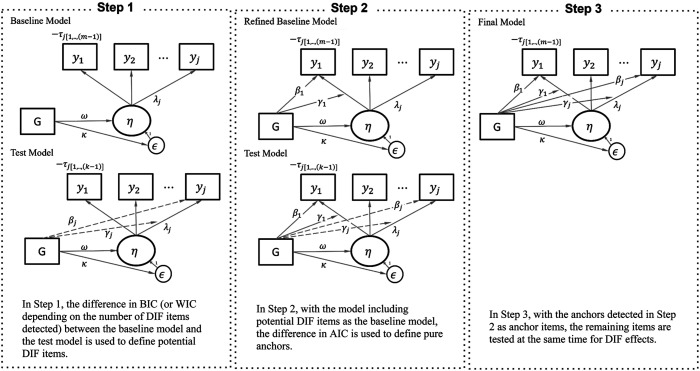
Conceptual Diagram for Refined Constrained-Baseline Approach Under MNLFA. *Note.* The intercept and residual of the latent factor are constrained to zero and one, respectively, for identification. The dashed line represents iteratively tested, while the solid line is estimated

The proposed approach is composed of three steps, as illustrated in Figure 1.

#### Step 1: Conservative DIF Screening

The process begins with fitting an MNLFA model that includes impact parameters in factor means and variances for all potential DIF sources. At this stage, all item parameters are constrained to be equal across groups (i.e., B and Γ **=** 0), forming the baseline model for all subsequent comparisons.

Following the baseline “no-DIF” model, we adopt an item-by-item testing approach. Each item is tested using a model that allows both uniform and non-uniform DIF effects from all covariates, while keeping all other items constrained. Rather than using traditional likelihood ratio tests (LRT), model comparisons are based on differences in IC between the baseline model and each test model. These differences are evaluated using a robust residual approach that converts them into standardized Z-statistics. Specifically:

(1) Calculate the IC difference: $\Delta IC_{j}=IC_{baseline}-IC_{test_{j}}$ .
(2) Apply the robust residual approach: $\Delta IC_{j}=\beta_{0}+e_{j}$ , where $\beta_{0}$ is assumed to be zero, representing the ideal scenario where there is no systematic difference between the baseline and test models. Under this assumption, $e_{j}=\Delta IC_{j}$ .
(3) Compute the robust standard error using the sandwich estimator: $SE_{robust}=\sqrt{{\left(T^{\prime }T\right)}^{-1}\left(T^{\prime }diag\left(e_{j}^{2}\right)T\right){\left(T^{\prime }T\right)}^{-1}}$ , where T is a design matrix^3^ and $e_{j}$ are the residuals (equal to $\Delta IC_{j}$ under $\beta_{0}$ = 0 assumption).
(4) Calculate the robust Z-statistic: $z_{j}=\frac{e_{j}}{SE_{robust}}$ .

Items are flagged as potential DIF when $z_{j}$ >1.65, corresponding to a one-tailed 95% confidence level. DIF identification follows a progressive screening process, beginning with the conservative BIC. This choice reflects BIC’s strengths in controlling Type I error and its consistency in model selection, particularly valuable in DIF detection, where accurately identifying true DIF effects while minimizing spurious effects.

When BIC’s conservative nature yields no DIF detection, often due to its stringent penalties, we use WIC, which is formulated as:

(9) WIC=λAIC+(1−λ)BIC,0≤λ≤1

where λ ranges from 0 to 1, representing the relative contribution of AIC versus BIC. Starting from λ = 0.1 and potentially increasing to λ = 0.9, this progressive weighting scheme gradually shifts from BIC- to AIC-dominance, relaxing conservatism while still controlling Type I errors. This progressive approach allows for a balanced evaluation of model fit and complexity.

#### Step 2: Confirm Anchor Items

The second step refines the initial DIF detection and confirms anchor items. We update the baseline model to include all Step 1 DIF candidates, then re-test the remaining unflagged items using the robust residual approach to assess IC differences. At this stage, we use AIC for a different purpose than in Step 1—prioritizing efficiency and predictive accuracy. Unlike BIC, which targets the true model, AIC seeks the model with the best approximation, making it well-suited for confirming anchor items based on goodness-of-fit and prediction rather than strict model correctness. In other words, because AIC favors more complex models and is prone to flagging DIF, items that remain unflagged can be confidently treated as “*pure*” anchors—strongly suggesting invariance across DIF sources. Items flagged in either Step 1 or 2 are carried forward for formal DIF testing in the next step.

#### Step 3: Final MNLFA With Selected Anchors

In the final step, we estimate the full MNLFA model using the confirmed anchor structure. Anchor items confirmed in Step 2 are constrained to be DIF-free, while remaining items are estimated with both uniform and non-uniform DIF across covariates. This yields a comprehensive model that captures DIF while preserving measurement invariance through anchor items.

This refined approach balances Type I and II error risks through three strategies: (1) initiating detection with conservative BIC, (2) using WIC when BIC is too strict, and (3) re-testing unflagged items to finalize anchors. BIC helps exclude spurious DIF, while AIC later confirms truly DIF-free items—leveraging their trade-off in model selection.

The method improves DIF detection accuracy and efficiency in MNLFA, addressing computational constraints and reducing Type I error inflation. To our knowledge, this is the first study to evaluate MNLFA-based anchor detection alongside DIF testing, offering a novel contribution to measurement modeling.

## Simulation Study

We conducted a Monte Carlo simulation to assess the performance of the refined constrained-baseline approach for anchor selection and DIF detection within the MNLFA framework. Key parameters were varied to test robustness under different conditions (Table 1).

**Table 1.** Description of the Simulation Conditions

	Conditions	Values
Manipulated	Total sample size	500, 1000, 2000
	Number of items	10, 20
	Proportions of anchor	40%, 70%
	DIF effect size	0.5 for uniform, 0.3 for non-uniform
	DIF scenarios	Two DIF, one DIF, empirical
Fixed	Number of DIF sources	3 (X1, X2, X3)
	Number of categories	4
	Coefficient for latent means and variances	[–0.25, −0.1, 0.25]/[0.1, 0.3, −0.3]

### Simulation Designs

All items followed a GRM with four categories (0–3). DIF was introduced via three covariates—two binary, one continuous—designed to mirror the covariance structure observed in the empirical dataset. Covariate effects on factor means (**Ω**) were set at −0.25, −0.10, and 0.25; effects on variances (**Κ**) at 0.1, 0.3, and −0.3. Thresholds were drawn from distributions centered at 0, 1.5, and 3.5; slopes from a uniform distribution between 2.0 and 4.0, aligning with typical psychological measures (e.g., PROMIS; Cella et al., 2019). The resulting item responses produced the skewed item response patterns were also consistent with the empirical data.

Five conditions were manipulated: sample sizes (500, 1000, 2000), item counts (10 or 20), and anchor proportions (40% or 70%). Remaining items exhibited both uniform and non-uniform DIF. DIF magnitudes reflected small (0.3 logits) and moderate (0.5 logits) effects.^4^ For uniform DIF, moderate effects were applied, while for non-uniform DIF, small DIF effects were used.

Three DIF scenarios were modeled. Scenario 1 (Two-DIF) included all three DIF sources: one item with uniform DIF, one with non-uniform, and one with both. Scenario 2 (One-DIF) had a single DIF source with all other items DIF-free. Scenario 3 (Empirical) applied both DIF types from distinct sources on each item. Detailed DIF settings are shown in Table 2.

**Table 2.** Detailed DIF Scenarios

Scenario		Uniform DIF effect			Non-uniform DIF effect		
		X1	X2	X3	X1	X2	X3
1	Item 8	0.5	−0.5	0.0	0.0	−0.3	0.3
	Item 9	−0.5	0.0	0.5	**−**0.3	0.0	0.3
	Item 10	0.0	0.5	−0.5	−0.3	0.3	0.0
2	Item 8	0.5	0.0	0.0	0.0	0.0	0.3
	Item 9	0.0	−0.5	0.0	0.0	−0.3	0.0
	Item 10	0.0	0.0	0.5	0.3	0.0	0.0
3	Item 8	0.3	−0.4	0.0	0.0	0.0	0.0
	Item 9	0.0	−0.4	−0.5	−0.5	0.0	0.5
	Item 10	−0.2	0.8	−0.3	0.0	−0.5	0.3

*Note.* Anchor proportion = 70% with 10 items. The same pattern applies to anchor proportion = 30% and 20 items; Scenario 1: two DIF per item; Scenario 2: one DIF per item; Scenario 3: empirically driven.

### Data Generation

Data were simulated in R (R Core Team, 2024) using a MNLFA framework with three covariates (X1–X3) as DIF sources. Covariate values were randomly drawn to match empirical statistics (see *Empirical Illustration*), and person parameters were sampled from normal distributions with specified impact means and variances as in Table 1. Responses were generated under a GRM, conditioned on item parameters and scenario-specific DIF effects. Each condition included 100 replications.

### Analysis Details

This study implements the refined constrained-baseline approach for anchor identification and DIF testing through a three-step process. Step 1 involved screening DIF via a constrained-baseline model. Robust Z statistics based on BIC differences were computed, with a conservative threshold (Z >1.65). If no items passed, WIC was applied with gradually increasing λ values (0.1 to 0.9) until DIF items emerged. In Step 2, DIF candidates identified in Step 1 were added to the baseline model. The remaining items were re-evaluated using AIC differences and robust Z statistics. Items showing no significant DIF were designated as *“pure”* anchor items. Step 3 estimated the final MNLFA model, including all DIF items and using the identified anchors from Step 2 for identification. DIF was flagged when covariate effects were significant (*p* < .05), indicating uniform, non-uniform, or both. Factor mean and residual variance were fixed at 0 and 1, respectively; thresholds and loadings were freely estimated. All models were estimated in M*plus* using a logit link, with convergence evaluated under the default EM algorithm criteria in M*plus* (see Chapter 16, Analysis Command options, Muthén & Muthén, 1998–2018).

To benchmark our approach, we used a regularization-based DIF detection method using the *regDIF* package (Belzak, 2023). Given the computational burden of latent scores in polytomous data, we used proxy scores (sum of item responses), which, despite bias (Belzak, 2023), have shown validity in DIF detection (Cardwell et al., 2022; Magis et al., 2015). Regularization proceeded in two steps. First, we estimated 100 models with decreasing tuning parameter values, starting with a large value (removing all DIF) and gradually permitting DIF effects.^5^ The best-fitting model via BIC identifies specific DIF effects for each item (Step 1). Next, the flagged DIF effects were re-estimated in M*plus* to assess their statistical significance (Step 2; Belzak & Bauer, 2020).

### Evaluation Criteria

The proposed method was compared to the regularization approach across anchor item selection (Steps 1–2) and final DIF estimation. Performance was assessed using the following metrics: Table 3 outlines the evaluation metrics.

**Table 3.** Confusion Matrix of Evaluation Criteria

	True DIF	True anchor
Flagged DIF	Power (correctly identifying true DIF at the final stage)	Type I error (incorrectly flagging DIF at the final stage)
Flagged anchor	Type II error (failing to identify items as potential DIF before the final stage)	Hit rate (correctly identifying items as “*pure*” anchors before the final stage)

*Note.* Rows represent model classifications (flagged as DIF or anchor), and columns represent the true data-generating status.

#### Step 1–2 Evaluation: Anchor Detection

*Hit Rate* is defined as the proportion of DIF-free items correctly classified as “*pure*” anchors prior to the final step. A “*pure*” anchor refers to an item for which no effects from any DIF source are flagged. This metric evaluates how effectively each method isolates items unaffected by DIF prior to the final estimation step. While no universal standard exists, we applied the same criteria used for power: hit rates above .80 are generally considered acceptable.

*Type II error* is defined the proportion of true DIF items mistakenly labeled as anchors—either in Step 2 (refined) or Step 1 (regularization). This misclassification excludes DIF items from the final estimation, risking bias in other model parameters. Because desired statistical power is typically set at 0.7–0.8 (Cohen, 1992), the corresponding Type II error threshold is 0.20. Accordingly, we regard 0.2–0.3 as tolerable, though lower values are preferred in DIF contexts, since high Type II error carries the risk of entirely excluding potential DIF items from the final model.

#### Step 3 Evaluation: Final DIF Estimation

*Type I Error* is the proportion of DIF-free effects wrongly flagged as significant. It includes: (1) DIF-free effects within DIF items tested in Step 3, and (2) “*pure*” anchors designated in Step 2, which by definition were excluded from the final model. As per Bradley (1978), acceptable Type I error ranges between 0.025 and 0.075 at α = 0.05.

*Power* is defined as the proportion of true DIF effects correctly identified in the final model—Step 3 for the refined approach and Step 2 for regularization. Power above 80% is considered excellent, while 70–80% is moderate (Cohen, 1992).

*Parameter Estimation Accuracy* is assessed via relative bias, defined as the difference between the mean estimated DIF effects and the corresponding true DIF value, divided by the true value. Only true DIF effects (uniform and non-uniform) from the three sources were evaluated; anchor items were not considered (true DIF = 0). We used the absolute value of bias, with values below 0.10–0.15 considered acceptable (Muthén et al., 1987), indicating higher accuracy at lower levels.

### Simulation Results

#### Hit Rate

**Figure 2.**
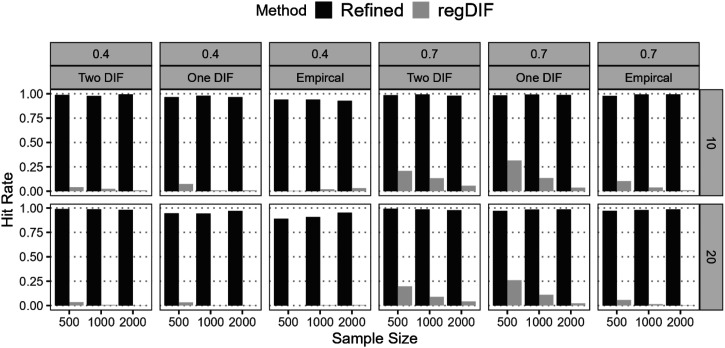
Hit Rates for Pure Anchors Before The Final Stages for Refined Constrained-Baseline and Regularization Methods

Figure 2 shows hit rates for correctly identifying “*pure*” anchors across varying conditions. The refined constrained-baseline approach achieved near-perfect rates in all cases, demonstrating high accuracy. By contrast, the regularization approach produced much lower rates, reflecting weaker anchor detection. This difference arises from how DIF is tested: regularization evaluates individual covariate effects, and an item is removed from the anchor set if any single effect is flagged, whereas the refined method evaluates DIF at the item level—all potential DIF sources are tested simultaneously for each item. Once selected, it is fully treated as DIF-free, reducing unnecessary inclusions. These findings highlight the refined approach’s superiority in reliably identifying stable anchors, essential for minimizing bias in later DIF detection.^6^

#### Type II Error

**Figure 3.**
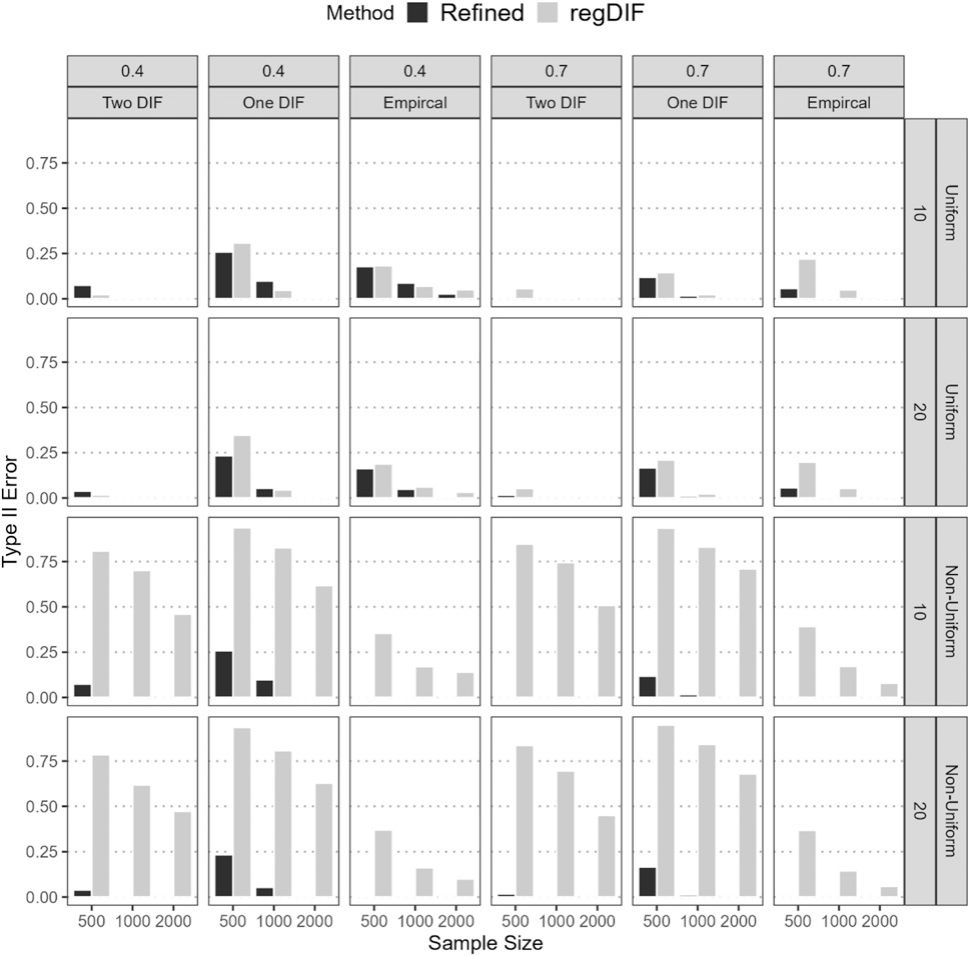
Type II Error for Not Detecting DIF of Refined Constrained-Baseline and Regularization Methods From the First Stage

Figure 3 presents Type II error rates for the refined constrained-baseline and regularization approaches across varying conditions. The refined method showed substantial improvement in controlling Type II errors under both uniform and non-uniform DIF. For uniform DIF, it maintained low error rates, particularly as sample size and anchor proportion increased (<0.3). Although the regularization method performed similarly for uniform DIF, it produced higher errors with smaller samples or lower anchor proportions. The most pronounced differences appeared in non-uniform DIF detection: the refined method consistently kept Type II error near zero except a few (*N* = 500, One-DIF), whereas the regularization method often failed. Under non-uniform One- or Two-DIF conditions with 500–1000 examinees, the regularization method’s error frequently exceeded 0.80 and sometimes approached 0.95. By contrast, the refined method rarely rose above 0.05, demonstrating superior performance in minimizing Type II errors, which means the low risk of mistakenly treating DIF items as anchors in the final model.

#### Type I Error

**Figure 4.**
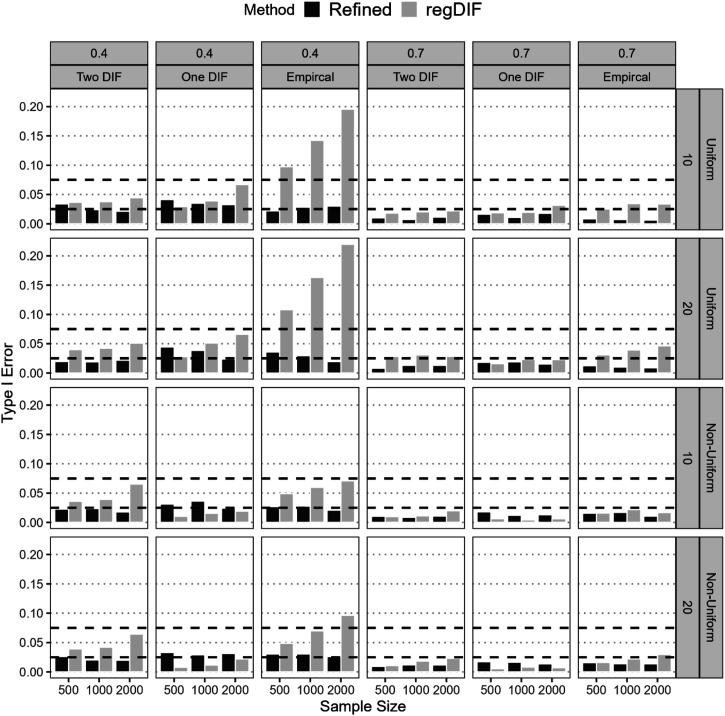
Type I Error of Refined Constrained-Baseline and Regularization methods. *Note*. All DIF-free effects that were initially flagged for DIF before the final step and subsequently excluded in the final model were included in the computation of Type I error

The refined method consistently controlled Type I error across all scenarios, remaining within the acceptable 0.025–0.075 range. For both uniform and non-uniform DIF, its error generally fell between 0.01 and 0.04, with only modest variation by sample size, anchor proportion, and item count. In contrast, the regularization method often produced inflated Type I errors, especially under low anchor conditions for uniform DIF. For instance, with *N* = 2,000 and 20 items, its error rose to 0.22. Even in more moderate cases (e.g., *N* = 1,000, 10 items, 40% anchors), it often exceeded 0.10, particularly under empirical DIF. Although regularization occasionally yielded acceptable Type I error in One- or Two-DIF conditions for non-uniform DIF, it failed in several conditions. Notably, under the empirical DIF scenario with *N* = 2,000, 40% anchors, and 20 items, inflated error was again observed. By contrast, the refined method consistently maintained well-controlled Type I error across all non-uniform DIF conditions (Figure 4).

#### Power

**Figure 5.**
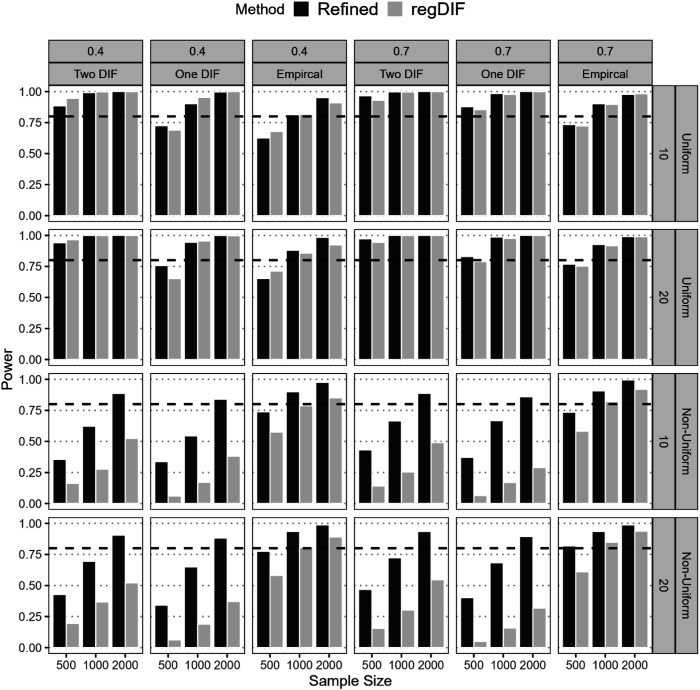
Power of DIF Detection of Refined Constrained-Baseline and Regularization methods. *Note*. All DIF items that were not initially flagged as DIF in the first step—and were therefore excluded from the final model—were treated as undetected DIF effects in the power computation

Figure 5 presents the power rates of the two approaches across various conditions. Overall, both methods showed high power for uniform DIF, particularly under favorable conditions. With moderate to large sample sizes (*N* = 1,000 or 2,000) and 70% anchor items, both achieved near-perfect power (≥0.99) in detecting uniform DIF across all scenarios. However, under constrained settings (*N* = 500, 40% anchors), power dropped below 0.8 for both, especially in One-DIF and Two-DIF scenarios. Performance diverged more sharply for non-uniform DIF. The refined method consistently outperformed regularization, reaching 0.8 power at *N* = 2,000 across all conditions. Regularization, by contrast, only achieved similar power in the empirical DIF scenario at *N* = 2,000. This gap aligns with the regularization method’s higher Type II error rates in these contexts, explaining its reduced effectiveness. Notably, the DIF effect size was 0.3 under which the refined method remained robust, while regularization struggled, especially in non-uniform DIF detection.

#### Accuracy of DIF Effects

**Figure 6.**
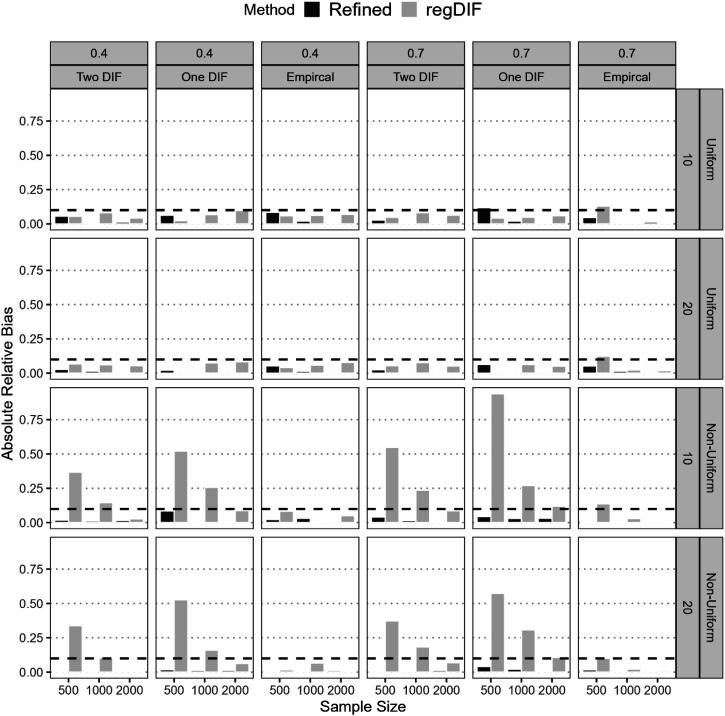
Relative Bias of DIF Detection of Refined Constrained-Baseline and Regularization Methods

Figure 6 presents the estimation accuracy of uniform and non-uniform DIF effects across conditions. The refined constrained-baseline method consistently produced low absolute relative bias, with nearly all values well below the 0.10–0.15 threshold for acceptable bias. Its accuracy held steady even under small samples and low anchor proportions, across both DIF types. In contrast, the regularization method showed substantial bias, especially for non-uniform DIF under One- and Two-DIF scenarios. For instance, in the One-DIF condition with *N* = 500 and 70% anchors, regularization yielded bias exceeding 0.90 for non-uniform DIF, compared to just 0.04 for the refined method. Across *N* = 500–1,000, regularization bias ranged from 0.15 to over 0.50, while the refined method remained within 0.001–0.04. Even in uniform DIF settings, regularization occasionally approached the upper bias limit (e.g., 0.12 and 0.13), whereas the refined method stayed below 0.10 in all but one case—One-DIF with *N* = 500, 70% anchors, and 10 items, where it reached 0.11.

## Empirical Illustrations

For illustrative purposes, and to evaluate the proposed approach under real-world conditions, we applied it within the MNLFA framework using empirical data. As a benchmark, we also implemented the regularization method. This comparison allowed us to examine each method’s effectiveness in identifying anchor items and DIF effects. It is important to note that this analysis is intended as a methodological demonstration—not as a basis for substantive claims about the population. The goal is to illustrate how the refined method is applied in practice.

### Data Illustration

This study uses data from the National Longitudinal Study of Youth 1997 (NLSY97), which tracked 8,984 U.S. adolescents born between 1980 and 1984. We focus on depressive symptoms measured in 2019 using the seven-item CES-D scale, rated on a 4-point Likert scale, with a final analytic sample of 3,391 participants. Three covariates were used as DIF sources: sex, race, and household poverty ratio. Sex was coded as 0 for male and 1 for female (50.9% male), and race was coded as 0 for non-Black/non-Hispanic and 1 for Black/Hispanic (48.8% Black/Hispanic). Poverty ratio was standardized using the 2019 household poverty ratio, with higher values indicating greater poverty. The correlations among the covariables were $\left(\begin{array}{ccc} 1 & & \\ 0.065 & 1 & \\ -0.041 & -0.219 & 1 \end{array}\right)$ . All data and analysis code used in this study are openly accessible on https://osf.io/mzdxh/.

### Analysis Details

The empirical study implements the same procedure as in the simulation study, where the refined constrained-baseline approach and regularization approach were used for DIF detection.^7^

### Empirical Results

Tables 4–6 summarize DIF and anchor item detection outcomes from both methods.

**Table 4.** Selected Possible DIF Candidate in Step 1 and Anchor Items in Step 2 for the Refined Constrained-Baseline Method

Item	Step1		Step2	
	BIC	ΔBIC	AIC	ΔAIC
1	29760.03	−42.454	**29363.62**	**−3.919**
2	29751.19	−33.606	**29352.11**	**7.594**
3	29712.35	5.227	29342.30	17.406
4	29742.30	−24.724	29346.22	13.485
5	**29604.86**	**112.721**		
6	29745.84	−28.259	**29365.26**	**−5.554**
7	29741.98	−24.397	29340.52	19.183

*Note.* Numbers in bold and gray indicate items selected as DIF candidates and “*pure*” anchor items in Step 1 and Step 2, respectively. In the regularization method, Item 2–7 each exhibit at least one DIF effect, which were included in the final model for DIF testing as seen in Tables 4 and 5.

**Table 5.** Tests for Uniform-DIF in Comparison Between Refined Constrained-Baseline and Regularization Methods

	Constrained-baseline			Regularization		
DIF	Est.	SE	*p*	Est.	SE	*p*
R3←POVERTY	0.028	0.075	0.706	0.060	0.061	0.326
R3←RACE	**−0.414**	**0.175**	**0.018**	**−0.464**	**0.127**	**0.000**
R3←SEX	**0.321**	**0.162**	**0.047**	**0.264**	**0.109**	**0.016**
R4←POVERTY	−0.559	0.303	0.065			
R4←RACE	−0.436	0.581	0.453	**−0.616**	**0.288**	**0.032**
R4←SEX	0.001	0.563	0.998	0.098	0.549	0.859
R5←POVERTY	**−0.336**	**0.097**	**0.001**	**−0.312**	**0.089**	**0.000**
R5←RACE	**1.045**	**0.116**	**0.000**	**1.025**	**0.117**	**0.000**
R5←SEX	−0.209	0.111	0.059	−0.211	0.110	0.055
R6←RACE				−0.051	0.099	0.608
R7←POVERTY	−0.028	0.154	0.854			
R7←RACE	**0.877**	**0.319**	**0.006**	**0.767**	**0.312**	**0.014**
R7←SEX	**0.895**	**0.332**	**0.007**	**0.893**	**0.333**	**0.007**

*Note.* Numbers in bold and gray indicate items flagged as DIF. Empty cells represent DIF-free items excluded in the final DIF testing model. R3–R7 represent items tested for uniform DIF, and the arrow (←) means the tested DIF effect from corresponding covariates.

**Table 6.** Tests for Non-Uniform-DIF in Comparison Between Refined Constrained-Baseline and Regularization Methods

	Constrained-baseline			Regularization		
DIF	Est.	SE	*p*	Est.	SE	*p*
R2←RACE				0.073	0.113	0.520
R3←POVERTY	0.110	0.079	0.163	0.039	0.065	0.549
R3←RACE	−0.030	0.160	0.852			
R3←SEX	−0.074	0.151	0.623			
R4←POVERTY	**0.641**	**0.266**	**0.016**			
R4←RACE	−0.088	0.447	0.844			
R4←SEX	−0.524	0.454	0.248	−0.587	0.450	0.192
R5←POVERTY	**0.345**	**0.090**	**0.000**	**0.277**	**0.083**	**0.001**
R5←RACE	**−0.496**	**0.104**	**0.000**	**−0.496**	**0.099**	**0.000**
R5←SEX	−0.091	0.100	0.365	−0.086	0.101	0.393
R6←POVERTY				0.000	0.045	0.995
R6←SEX				0.028	0.089	0.757
R7←POVERTY	**0.306**	**0.156**	**0.049**	**0.188**	**0.062**	**0.002**
R7←RACE	**−0.732**	**0.271**	**0.007**	**−0.667**	**0.241**	**0.006**
R7←SEX	**−0.736**	**0.285**	**0.010**	**−0.723**	**0.287**	**0.012**

*Note.* Numbers in bold and gray indicate items flagged as DIF. Empty cells represent DIF-free items that were not included in the final DIF testing model. R3–R7 represent items tested for non-uniform DIF, and the arrow (←) means the tested DIF effect from corresponding covariates.

#### Anchor Detection

Table 4 shows DIF and anchor item classification from Steps 1 and 2 (refined method) and anchor status based on regularization. The regularization method flags individual covariate effects; thus, items without significant effects are treated as “*pure”* anchors.

Both methods identified Item 1 as an anchor, though regularization required a follow-up significance test. The refined method flagged one DIF item in Step 1 and confirmed three anchor items in Step 2.

#### DIF Analysis

Tables 5 and 6 provide final DIF estimates. For the refined method, Step 3 results include all non-anchor items from Step 2. For regularization, flagged effects from Step 1 were tested. Table 5 compares uniform DIF results. Both methods agreed broadly, with one discrepancy: for Item 4, race-related DIF was non-significant using the refined method but significant via regularization.

Table 6 covers non-uniform DIF. Again, results were largely aligned except for one case: the refined method identified significant poverty-related DIF for Item 4, which the regularization method missed, as it was not flagged in Step 1. Since unflagged effects are excluded from further testing under regularization, this omission is likely to reflect a Type II error. This case underscores the value of triangulating findings and combining statistical analysis with expert judgment. Aside from this difference, both approaches yielded closely matching non-uniform DIF results.

## Discussion

This study contributes to methodological literature by introducing an efficient approach to detecting DIF within the MNLFA framework. The refined constrained-baseline method addresses a major gap in MNLFA by offering a systematic, accessible solution for anchor selection and DIF detection.

The simulation results consistently demonstrated the advantages of the refined constrained-baseline anchor detection method over the regularization approach. Most notably, the refined method achieved acceptable Type II error across all conditions, exhibiting exceptional sensitivity in detecting both uniform and non-uniform DIF, even under small sample sizes and complex DIF structures. It also maintained strict control over Type I error, especially under large sample and low-anchor-ratio conditions, outperforming the regularization method.

A noteworthy consideration is the source of the unusually low Type I error rates. This outcome stems from how Type I error was computed: rather than using only the DIF effects tested in the final step, the calculation also included “*pure*” anchor items that had already been flagged earlier in the process (i.e., Step 2). Because these anchor effects were excluded from the final step, they had no chance of being incorrectly classified as DIF. Given the high hit rates of the proposed method, the inclusion of these non-tested anchor effects contributed to the notably low Type I error rates.

The refined method also yielded more precise estimates for DIF effects, with consistently lower absolute relative bias, especially for non-uniform DIF under One-DIF scenarios, where regularization exhibited inflated bias. Moreover, the refined approach maintained higher power in detecting non-uniform DIF. These advantages affirm its strengths in preserving anchor set validity, maximizing detection power, and ensuring accurate DIF effects recovery across a range of DIF conditions.

The empirical illustration showed both similarities and differences between the refined constrained-baseline method and the regularization approach. While there was agreement in identifying one key anchor and several DIF effects, differences emerged—particularly around non-uniform DIF detection, where the regularization method showed a higher tendency toward Type II errors. These patterns were echoed in the simulation results: the refined approach consistently outperformed the regularization method in detecting DIF, especially for non-uniform effects.

### Limitations and Future Directions

Several limitations point to directions for future research. First, the conditions explored in this study are somewhat exploratory due to the novelty of the method. According to Paxton et al. (2001), future research should build on these findings, examine the conditions more systematically, and connect them to the literature on this emerging topic. Second, while the GRM was appropriate for the psychological constructs examined here, educational testing often relies on other models, such as the generalized partial credit model (GPCM). Future work should evaluate the performance of the refined approach with alternative response formats. Third, though applied within the MNLFA framework, the refined method could extend to other models, such as multiple-group SEM or MIMIC. Testing its performance across frameworks would enhance generalizability. Finally, our simulations were limited to tests with 10 or 20 items. Pilot runs with 30 items revealed very slow convergence mainly due to the growing number of moderated parameters. Future research could adopt Bayesian estimation, which improves convergence and supports model comparison tools like WAIC or DIC. Alternatively, simpler strategies—like logistic regression using latent scores from unconditional MNLFA—may offer efficient options for larger assessments.

In sum, this study introduced the refined constrained-baseline approach for anchor detection within MNLFA, demonstrating strong performance in identifying DIF in polytomous items. The method outperforms regularization in accuracy while offering practical advantages in computation and accessibility. However, it should be applied alongside content expertise and, where possible, complementary detection methods to ensure robust DIF assessment in psychological and educational contexts.

## Supplemental Material

**Supplemental Material -** Anchor Detection Strategy in Moderated Non-Linear Factor Analysis for Differential Item Functioning (DIF) Supplemental Material for Anchor Detection Strategy in Moderated Non-Linear Factor Analysis for Differential Item Functioning (DIF) by Sooyong Lee, Suyoung Kim, and Seung W. Choi in Applied Psychological Measurement.

**Supplemental Material -** Anchor Detection Strategy in Moderated Non-Linear Factor Analysis for Differential Item Functioning (DIF) Supplemental Material for Anchor Detection Strategy in Moderated Non-Linear Factor Analysis for Differential Item Functioning (DIF) by Sooyong Lee, Suyoung Kim, and Seung W. Choi in Applied Psychological Measurement.

## Notes

1.
$\tau_{jc}$ represents the log-odds of responding in the lower category. For example, in the binary case, $\log\left(\frac{P\left(y_{j}=0\right)}{P\left(y_{j}=1\right)}\right)=\tau_{jc}$ . Therefore, with the negative sign, $-\tau_{jc}$ corresponds to the log-odds of responding in the higher category: $\log\left(\frac{P\left(y_{j}=1\right)}{P\left(y_{j}=0\right)}\right)={-\tau }_{jc}$ .

2. It is important to note that the variance estimate must be positive, which is why the log-linear functions are employed for variances to obtain positive values.

3.
T is simply a J×1 matrix of ones, since the model includes only an intercept.

4. This criteria, so-called P2 scheme, is based on the ETS scheme for dichotomous items adjusted for graded response models. Penfield (2007) found in the pilot study 0.3 are classified into category A, 0.5 into category A and B, and 0.7 into B and C.

5. The package’s default setting was used. In addition to the default settings, we explored a wider range of penalty values in an attempt to mitigate the potential inflation of Type I error sometimes associated with regularization methods. However, the results remained largely consistent across these alternative settings. Consequently, we report the findings based on the default tuning parameter sequence.

6. We checked likelihood ratio test based on the typical constrained-baseline approach, but known as highly inflated Type I error. We also found the same pattern based on the MNLFA, which is seen in Figure 3 of the Online Supplemental materials.

7. We also applied a regularization method with latent traits under MNLFA (not shown). In Step 1, four potential DIF effects were identified: thresholds of item R3 by Race and Sex, and item R5 by Race and Poverty Ratio. No DIF appeared in item slopes. Compared to results from the refined constrained-baseline method and proxy-based regularization, these findings are overly conservative.
