# Supplementary material for: Anchor Detection Strategy in Moderated Non-Linear Factor Analysis for Differential Item Functioning (DIF)
Source: Appl Psychol Meas. 2025 Nov 24:01466216251401206. Online ahead of print. doi: 10.1177/01466216251401206 (PMC12643905; doi:10.1177/01466216251401206)

Supplementary Materials

1. Additional Type I Error Results by Anchor set and DIF set separately

Figure 1.

Type I Error within Anchor Set

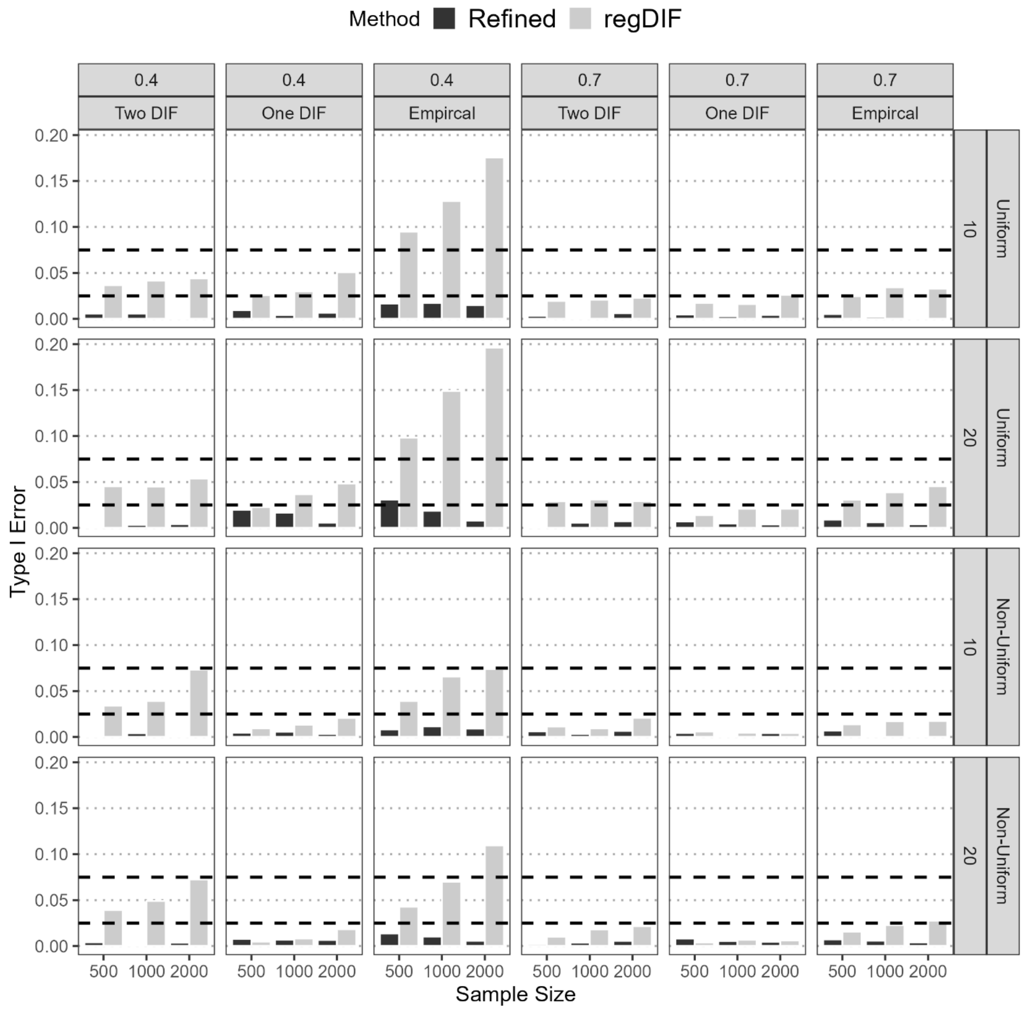

Note. Items flagged as “pure” anchors before the final step were treated as insignificant DIF effects, so that they are included when computing Type I error within a set of anchors.

Figure 2.

Type I Error within DIF Set

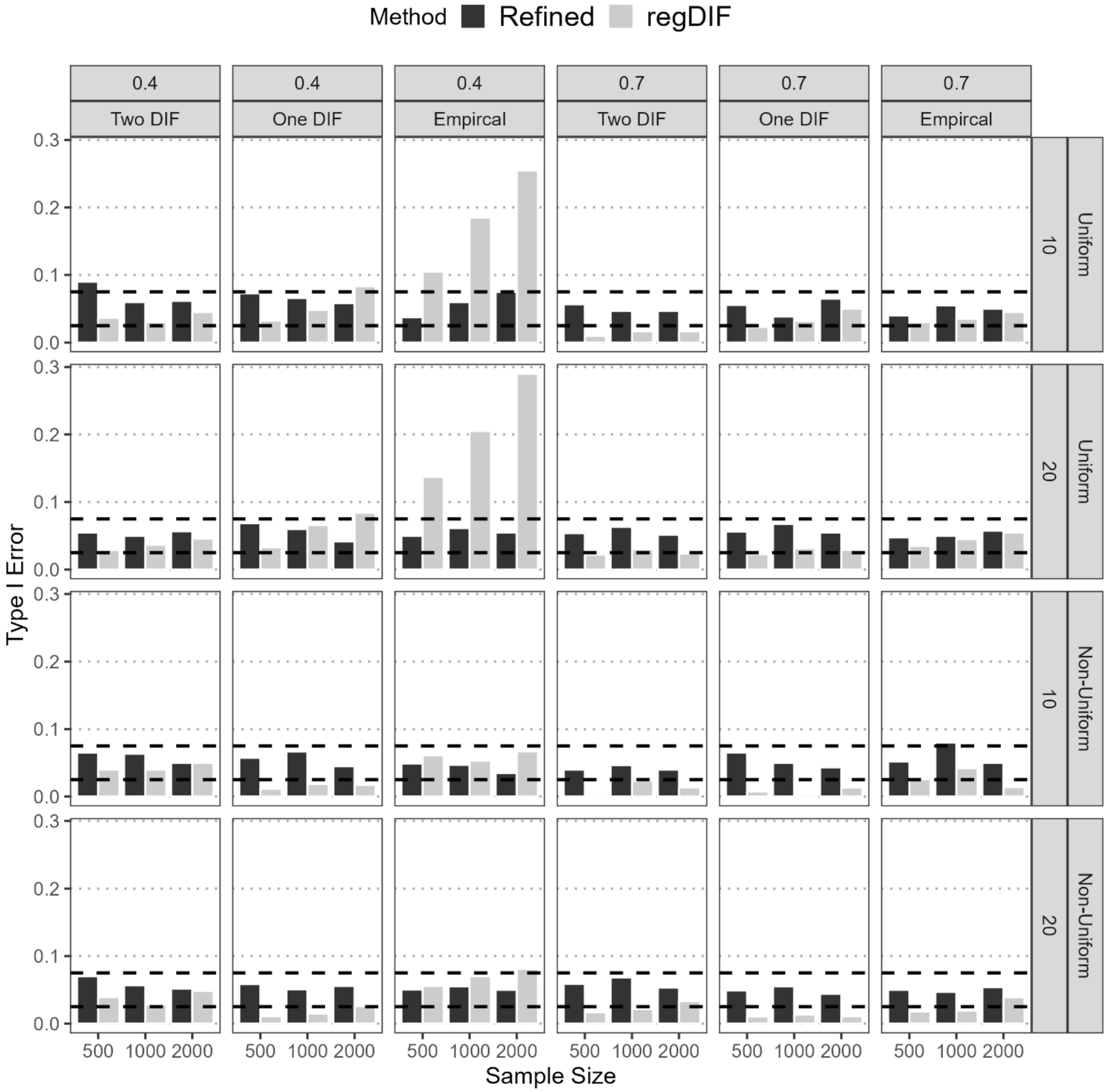

2. **Type I error with Likelihood Ratio Test.**

Figure 3.

Type I error of LRT method

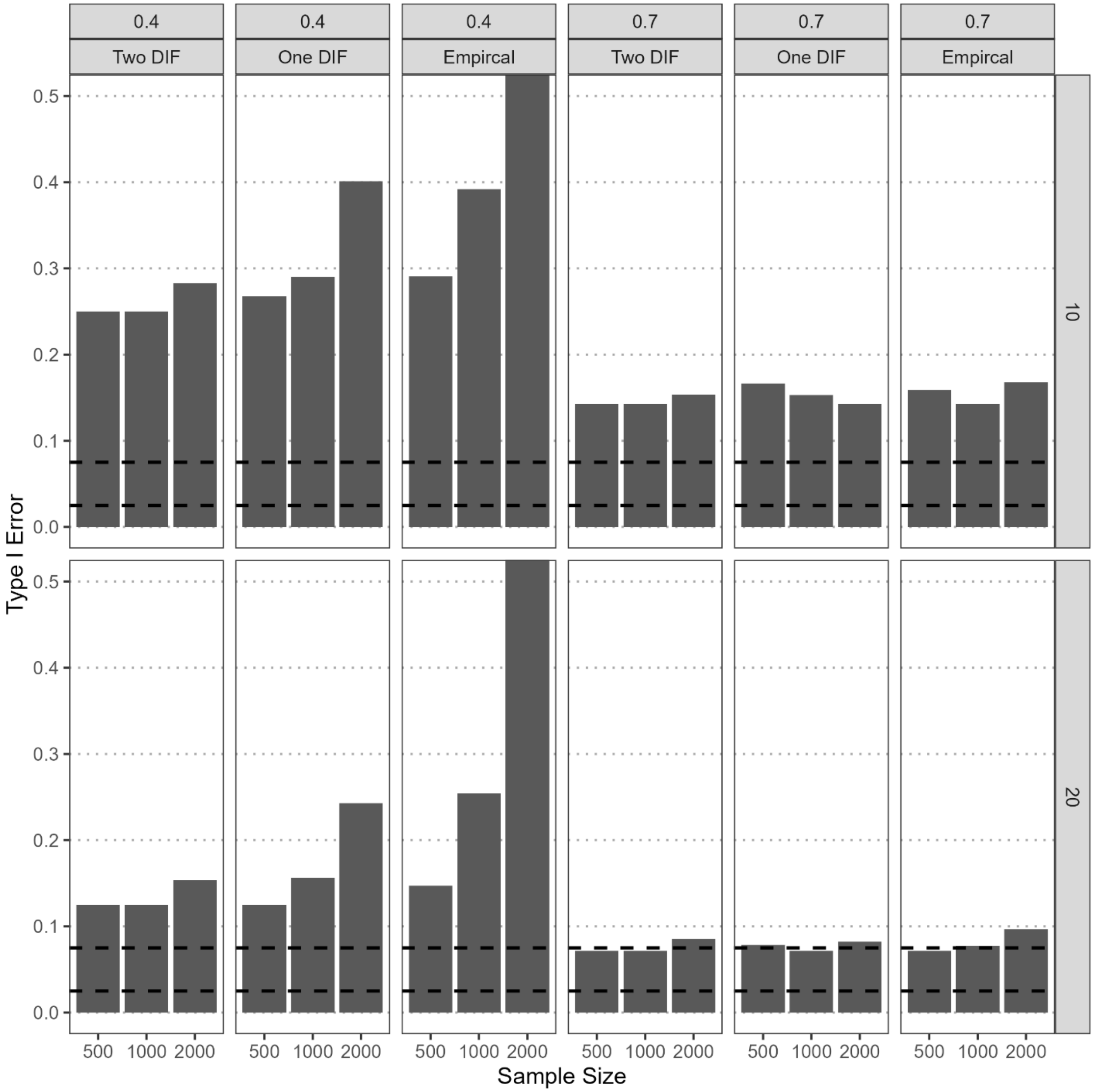

Supplement: Supplemental Material - Anchor Detection Strategy in Moderated Non-Linear Factor Analysis for Differential Item Functioning (DIF) [file sj-pdf-1-apm-10.1177_01466216251401206.pdf]
