# Supplementary material for: Anchor Detection Strategy in Moderated Non-Linear Factor Analysis for Differential Item Functioning (DIF)
Source: Appl Psychol Meas. 2025 Nov 24:01466216251401206. Online ahead of print. doi: 10.1177/01466216251401206 (PMC12643905; doi:10.1177/01466216251401206)

1. **Additional runs with 8000 sample sizes**

Figure 3

Type I error of refined constrained-baseline and regularization methods under 8000 sample sizes

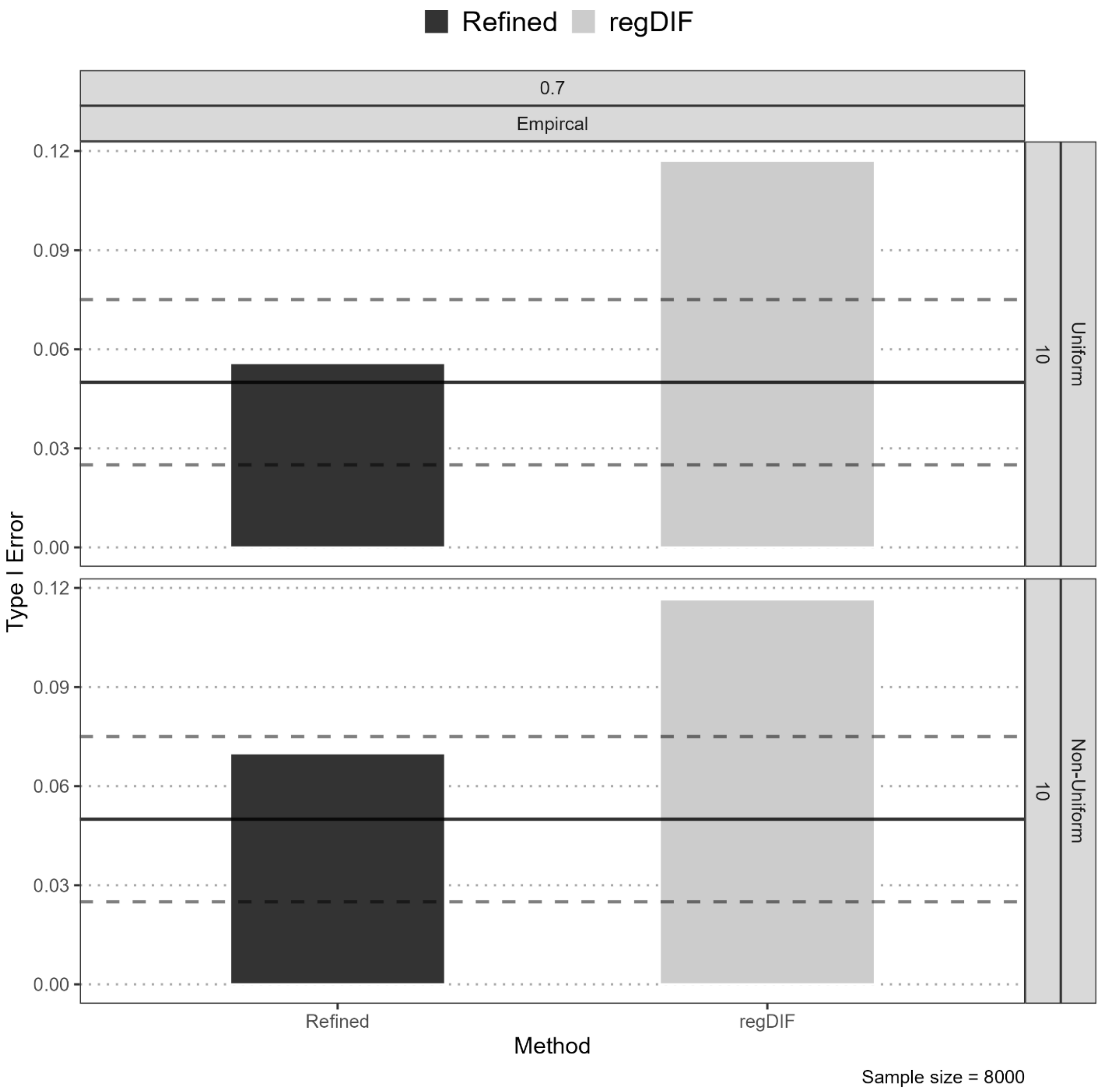

Note. All DIF-free effects that were initially flagged for DIF before the final step and subsequently excluded in the final model were included in the computation of Type I error.

Figure 4

Power of DIF detection of refined constrained-baseline and regularization methods under 8000 sample sizes

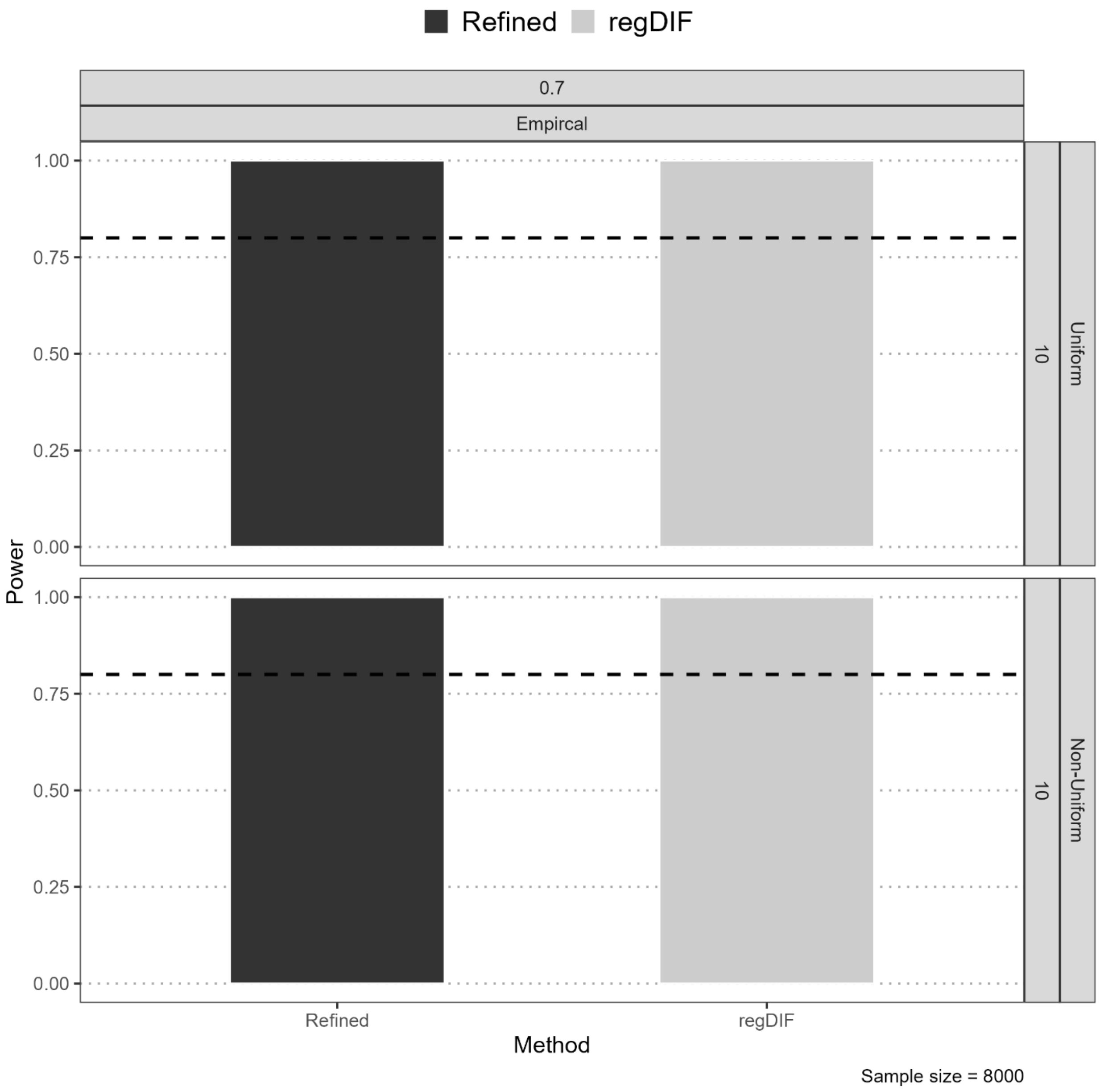

Note. All DIF items that were not initially flagged as DIF in the first step—and were therefore excluded from the final model—were treated as undetected DIF effects in the power computation.

Figure 5

Absolute Relative Bias of DIF effects of refined constrained-baseline and regularization methods under 8000 sample sizes

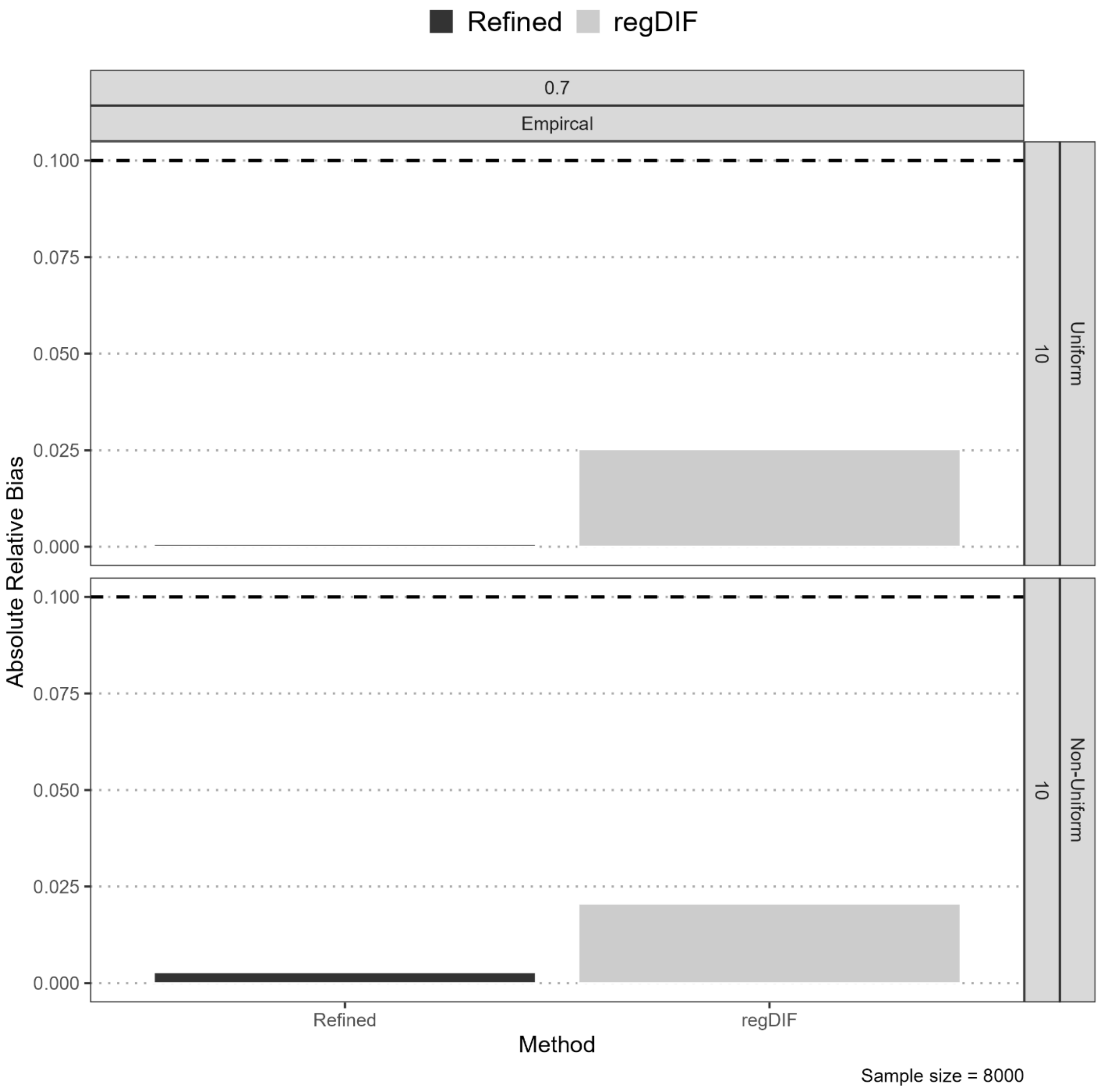

Supplement: Supplemental Material - Anchor Detection Strategy in Moderated Non-Linear Factor Analysis for Differential Item Functioning (DIF) [file sj-pdf-2-apm-10.1177_01466216251401206.pdf]
